# A framework for using magic to study the mind

**DOI:** 10.3389/fpsyg.2014.01508

**Published:** 2015-02-02

**Authors:** Ronald A. Rensink, Gustav Kuhn

**Affiliations:** ^1^Departments of Psychology and Computer Science, University of British ColumbiaVancouver, BC, Canada; ^2^Department of Psychology, Goldsmiths University of LondonLondon, UK

**Keywords:** attention, cognition, magic, methodology, neuroscience, visual perception, wonder

## Abstract

Over the centuries, magicians have developed extensive knowledge about the manipulation of the human mind—knowledge that has been largely ignored by psychology. It has recently been argued that this knowledge could help improve our understanding of human cognition and consciousness. But how might this be done? And how much could it ultimately contribute to the exploration of the human mind? We propose here a framework outlining how knowledge about magic can be used to help us understand the human mind. Various approaches—both old and new—are surveyed, in terms of four different levels. The first focuses on the *methods* in magic, using these to suggest new approaches to existing issues in psychology. The second focuses on the *effects* that magic can produce, such as the sense of wonder induced by seeing an apparently impossible event. Third is the consideration of *magic tricks*—methods and effects together—as phenomena of scientific interest in their own right. Finally, there is the organization of knowledge about magic into an *informative whole*, including the possibility of a science centered around the experience of wonder.

Magic is among the oldest of the performing arts. Given its concern with “creating illusions of the impossible” (Nelms, 1969/1996, p. 1), its practitioners—magicians—have had considerable opportunity to explore various ways of manipulating people’s thoughts, beliefs and perceptual experiences. The tricks and illusions resulting from this exploration are remarkably powerful, and have baﬄed audiences all over the world. They have also piqued the interest of scientists, including some of the earliest pioneers in psychology (see Hyman, 1989; Lamont, 2010). For example, Binet (1894) studied the techniques of several eminent magicians via the most careful measurements possible at that time. Among other things, he used the newly developed chronophotographic gun to investigate sleight of hand, discovering several of the perceptual mechanisms involved (see Lachapelle, 2008). Others, such as Triplett (1900), investigated specific magic illusions, and showed how just the suggestion of an action can trigger an illusory percept.

Although scientific interest in magic later waned, it never disappeared completely (see Hyman, 1989; Lamont and Wiseman, 1999); indeed, a new wave of interest has recently arisen. For instance, Kuhn and colleagues used eye tracking to explore our failure to see particular events during magic tricks (e.g., Kuhn and Tatler, 2005; Kuhn et al., 2009). Others investigated how misdirection (e.g., curved motion) can result in oculomotor behaviors that alter perception (Otero-Millan et al., 2011). Tracking of magicians’ eyes has revealed how social cues can drive our attention and perception (Kuhn et al., 2009; Cui et al., 2011). Additionally, investigations into the Indian rope trick have shown how memories of apparently impossible events can be related to memory distortions over time (Wiseman and Lamont, 1996; Wiseman and Greening, 2005).

Magic has also been used to investigate higher-level processes, such as belief formation and reasoning. For example, Benassi et al. (1980) showed that exposure to magic tricks (portrayed as demonstrations of psychic powers) increased belief in psychic phenomena (also see Mohr et al., 2014). Subbotsky (2010) used magic demonstrations to investigate magical thinking in both children and adults. Magic has even been used to explore the neural basis of causality (Parris et al., 2009), the origins of insightful thinking (Danek et al., 2013, 2014), and the nature of free will (Shalom et al., 2013).

Yet despite all this, research involving magic has remained scattered, with little or no attempt to connect the results of various studies, compare methodologies, suggest which new lines of research are promising, or determine how magic might best be used to study the human mind. It has recently been argued that it is time for scientists and magicians to study magic in a more scientific way, and develop connections to the other sciences involved with perception and cognition (Kuhn et al., 2008; Macknik et al., 2008). But how might this be done? And to what extent could magic ultimately contribute to our exploration of the human mind?

In this paper we propose a framework that describes many of the approaches that have been—or could be—taken to use magic to investigate human perception and cognition. This framework organizes these approaches into four different levels, ordered by the complexity of the issues involved. The first concerns adaptation of traditional magic techniques to help investigate current research issues. The second involves the nature of those effects that magic is uniquely suited for, such as the sense of wonder induced by an apparently impossible event. The third considers magic tricks as phenomena of scientific interest in their own right. The final level concerns the possibility of larger-scale patterns among magic tricks. We show that this framework cannot only collect and organize virtually all the work to date that has used magic to study the human mind, but also points toward a coherent program of research that could lead to interesting new avenues of research.

## APPLICATION OF MAGIC TECHNIQUES

Magicians have experimented with distorting reality for millennia (see Christopher and Christopher, 2006). They are not the only ones who do so: film directors, for instance, can manipulate our sense of time and space in ways that are often quite similar (Kinsley, 1993), and pickpockets can manipulate their victim’s tactile awareness using techniques that parallel those of the conjuror. Such convergences suggest that many of the techniques involved rely on perceptual and cognitive effects that are quite general.

Two aspects of a magic trick are of central importance. The first is the *effect—*the phenomena consciously experienced by the spectator (e.g., seeing a deck of cards riﬄed by a magician; seeing a chosen card emerge from the magician’s pocket). The second is the *method—*the manipulations used by the magician to achieve the effect (e.g., the particular way the cards are riﬄed; the placing of the card in the pocket ahead of time). In general, any effect can usually be produced by several different methods; conversely, any method can help create different effects (see, e.g., Tarbell, 1927/1971). Importantly, if a magic trick is to work, its method must be powerful enough to fool virtually an entire audience. As such, these methods—and their associated effects—could be harnessed to empirically investigate issues in perception, cognition, and other aspects of the human mind. Their applications can be readily grouped according to the perceptual and cognitive mechanisms involved.

### PERCEPTION

#### Object constancy

Developmental psychologists have long depended on magic methods for *conjuring*—making objects seem to disappear and reappear. For example, in the violation-of-expectation paradigm, the researcher may cover an object with a barrier, and then remove it to reveal that the object has disappeared; the assumption is that if infants have a sense of object constancy (i.e., objects continue to exist when out of sight), they should be surprised by the apparently impossible event. This paradigm has been used to investigate infants’ understanding of the physical world in general, ranging from the idea that objects cannot occupy the same space (penetration effect) to the concept that stable objects need a support of some kind (see Baillargeon, 1994). Related tricks have similarly been used to duplicate objects, allowing researchers to pretend they had a magical photocopy machine (Hood and Bloom, 2008).

Such techniques have also been used to investigate cognition in adults. For example, in a study on choice blindness (Johansson et al., 2005; Hall et al., 2010), participants were shown a pair of objects and asked to select the one they preferred. The selected object was then switched for the other one using a magic trick, so that this switch wasn’t noticed; participants then defended their “choice” by confabulating reasons why the switched object was superior to the originally selected one. The success of this approach relied on the conviction of the participants that the object could not have changed. While conventional techniques could have used images of objects on a computer screen, magic tricks allowed this to be done with physical objects, creating a much stronger belief that the object did not change, likely because there are far fewer ways for this to have occurred.

#### Visual attention

Another important aspect of magic is the control of visual attention, which determines what an observer consciously sees (Kuhn et al., 2008, 2014; Rensink, 2010, 2015). Various methods can be used for this. For instance, Kuhn et al. (2009) manipulated the direction of the magician’s gaze, influencing what participants saw. Another study found that individuals with autism were slower to fixate the face of the magician and less likely to follow gaze, suggesting that they were less efficient at using social cues (Kuhn et al., 2010). In both examples, magic provided a natural context in which to study these issues, without sacrificing any experimental control.

Many magic tricks use attentional misdirection to prevent an observer from detecting a visually salient event. This can be harnessed as well. For example, misdirection prevented participants from noticing a magician dropping a lighter onto his lap (Kuhn et al., 2008). The probability of noticing this was a natural measure of the effectiveness of the misdirection, allowing researchers to determine the effectiveness of different misdirection principles in controlling attention. (For a full review see Kuhn and Martinez, 2012).

Although several studies have investigated attentional control, only a small fraction of its potential has been explored to date. For example, researchers have largely ignored the influence of linguistic cues, although these can be readily studied (Teszka et al., 2010). Misdirection principles relating to body language and gesture likewise go beyond the issues generally investigated at present. Magicians also misdirect attention by using humor to create periods of attentional relaxation (e.g., Ortiz, 1994), another phenomenon apparently not yet investigated.

The experience of magicians shows that attention can also be controlled by factors at even higher levels of processing (Sharpe, 1988; Kuhn and Tatler, 2011; Kuhn and Martinez, 2012; Kuhn et al., 2014). For example, the *principle of naturalness* states that people are less suspicious of natural than unnatural actions, and so take less notice of the former (Sharpe, 1988). People likewise pay less attention to actions that are justified. Phenomena such as these are likely worth studying in a more rigorous way.

#### Expectation in vision

Although attention is an important factor governing what we consciously see, it is not the only one; another is *expectation* (e.g., Braun, 2001). This stems from the fact that much of perception must anticipate what will happen in the immediate future (Hawkins and Blakeslee, 2004), as well as compensate for processing delays (Cavanagh, 1997). Our conscious experience likely reflects the expectations created by these predictions.

**FIGURE 1:**
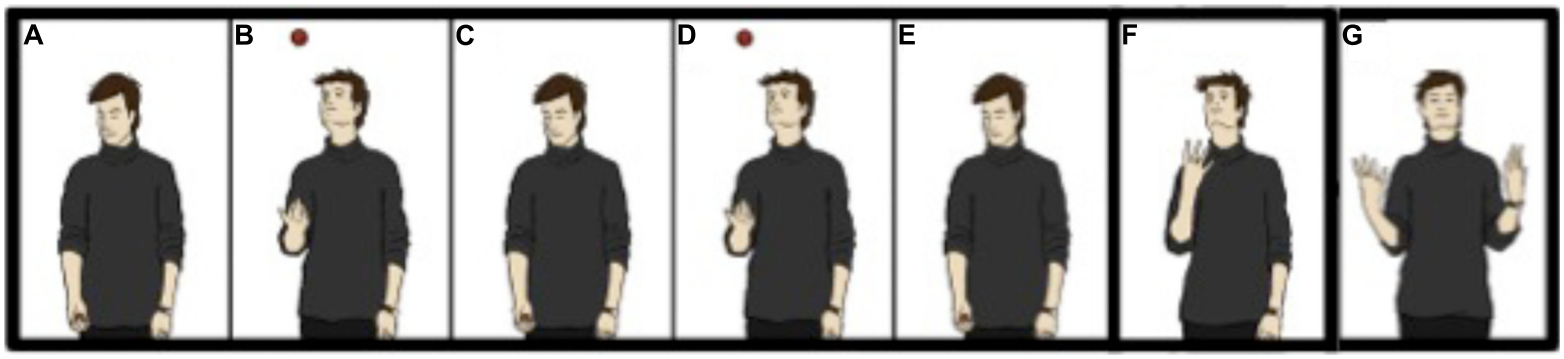
**The Vanishing Ball: (A) The magician is holding a small ball. (B–E)** He throws the ball in the air twice, following its trajectory with his gaze. **(F)** He pretends to throw the ball, but actually retains it in his hand; meanwhile, he looks up, following the expected trajectory of the ball. **(G)** The magician pretends that both hands are empty. The ball is typically seen as traveling upward and then vanishing. Figure from Friebertshauser et al. (2014).

The importance of expectation has been known to magicians for years. For example, in “The Vanishing Ball” (**Figure 1**), a ball seemingly vanishes while being thrown upward by the magician. This effect relies on the expectation that the ball actually is thrown upward (see, e.g., Triplett, 1900; Kuhn and Land, 2006); if this expectation exists, the observer will consciously experience the ball, even though no visual stimuli exist. Interestingly, the experience of the ball disappears while attention is being given to the illusory ball, indicating that attention alone cannot keep the underlying perceptual structures active. The methods used to create such vanishes could likely be adapted to explore these matters further—e.g., articulating the role played by expectation in visual experience, or perhaps mapping out the nature of the expectations themselves.

#### Visual illusions

Magic tricks often rely on illusions of various kinds (e.g., Sharpe, 1985). Many of these are based on well-known mechanisms, such as Gestalt laws of grouping, which can enable items to “disappear” via incorporation into larger-scale structures (Barnhart, 2010). However, some tricks use sophisticated methods that are not as well known. For instance, the techniques used in Pepper’s ghost illusion can make an object appear and disappear in full view of the spectator, or even seem to change into something else entirely (Christopher and Christopher, 2006). Such methods could be the basis for new kinds of investigation into visual perception.

### COGNITION

#### Hypothesis formation

A critical element of any magic trick is *misdirection*—manipulating the spectator away from the cause of the effect (e.g., Hugard, 1960; de Ascanio, 1964; Wonder and Minch, 1996; Crone, 1999; Kuhn and Martinez, 2012; Kuhn et al., 2014). This concept is a broad one, in that many kinds of mechanisms in the human mind are involved in making sense of incoming information. At the level of perception, misdirection often takes the form of attentional control (see Perception). But misdirection also applies to higher-level mechanisms, such as those enabling our understanding or memory of a situation (see Kuhn et al., 2014). Factors such as pre-existing knowledge and assumptions clearly play a role in this. Misdirection could help investigate how such factors interact.

Misdirection in the Vanishing Ball creates a hypothesis differing considerably from reality. It can likewise induce compelling—but untrue—explanations at higher levels (Lamont, 2013). Several interesting issues could be explored here. For example, why is a given explanation initially accepted over others that seem equally suitable? What counts as adequate evidence? Could several hypotheses be considered at the same time? Such methods might also help us understand phenomena such as confirmation bias, in which evidence supporting existing beliefs is favored in some way (Nickerson, 1998).

#### Memory

Conjurors often use suggestion to manipulate the spectator’s memories of a performance. A striking example of this can be found in eyewitness reports of the Indian rope trick, in which a magician levitates a long piece of rope, which an assistant then climbs. It is extremely unlikely that this trick was ever performed the way it is reported; instead, it appears to be a result of false memories (Wiseman and Lamont, 1996). More generally, memory distortions can prevent observers from recollecting a true sequence of events, and thus, from discovering the method behind an effect. This can be done in several ways: subtle details could be altered (e.g., forgetting or falsely remembering details that never took place), or the effect itself may be exaggerated (e.g., stating that five rather than three lemons appeared under the cup).

Wiseman and Greening (2005) investigated how recollection of an event could be influenced by such suggestions. Participants watched a video of a magician performing a psychokinetic key-bending trick. After the key-bending was completed and the bent key placed on the table, half the participants were given an additional suggestion implying that the key was still bending. Participants who received this suggestion were more likely to report having seen the key bend on the table.

These kinds of manipulations are extremely powerful; controlled investigation based upon them could therefore shed interesting new light on the mechanisms underlying memory. Among other things, they may reveal interesting individual differences by which memory distortions occur. As such, they may also have important practical applications—for example, highlighting limitations in the reliability of eyewitness testimonies.

#### Problem solving

Although magicians often go to great lengths to prevent people from discovering a method, this still happens on occasion. When it does, the trick fails. This is often accompanied by an *Aha* experience, a strong feeling that a solution has been found, along with a certainty that this solution is correct. This is an example of *insight* (Sternberg and Davidson, 1995; Bowden et al., 2005).

Insightful problem solving has typically been investigated using verbal problems or simple puzzles (Knoblich et al., 1999). However, such tasks are sometimes considered overly restricted (MacGregor and Cunningham, 2008). A possible way around this was proposed by Danek et al. (2013, 2014), who developed a battery of magic tricks for which the method could be discovered relatively easily. Participants watched videos of these tricks and tried to discover how they were done. Correct solutions were accompanied by an Aha experience almost 40% of the time, suggesting they were found through insightful problem solving.

Danek et al. (2013, 2014) argue that this approach offers several advantages over traditional ones (see also Demacheva et al., 2012). Among other things, they find that participants in their experiments are highly motivated to find the correct solution—once most people have observed a magic trick, they strongly wish to know how it was done. This motivation may be due to the experience of a phenomenon violating expectations about how the world works (e.g., seeing an object suddenly vanish), something not characteristic of verbal material or puzzles. It would be interesting to see whether this is also true outside the lab; according to Ortiz (1994), magic and puzzle-solving differ precisely along the dimension of emotional engagement, regardless of location. It would also be interesting to see if the attitudes of magicians to puzzle-solving are similar to those of non-magicians.

### OTHER

#### Agency and free will

We generally feel we have control over the decisions and choices we make. But the extent to which we actually control our behavior has been heavily debated. Studies have shown that behavior can be influenced by subliminal (unseen) cues (e.g., Lau and Passingham, 2007). But while such findings are reliable, the effects tend to be small, and so they are often discounted.

Meanwhile, influencing choice without the awareness of the influence—an effect known as *forcing*—is a major part of conjuring (Sharpe, 1988). For example, a magician may ask you to select a card. Although you may feel that your selection was a free one (i.e., a one in 52 chance of selecting that particular card), it was in fact largely predetermined (see, e.g., Kuhn et al., 2008).

In contrast to the relatively small effects created by subliminal cues, effects due to forcing can be quite large. For instance, Olson et al. (2013) and Shalom et al. (2013) used a popular forcing technique based on the duration the cards are shown. Results showed that the choice of card could be strongly affected, even when participants were unaware of the manipulation. These effects are large enough to potentially have applications in the real world (e.g., advertising).

#### Motor skills

Skilled magicians spend hours practicing methods such as sleight of hand (Rissanen et al., 2013, 2014). This has much in common with practicing an instrument: countless hours are devoted to rehearsing particular movement patterns. Much of what we know about skill acquisition is obtained from studying expertise in domains ranging from sports to chess (Didierjean and Gobet, 2008). The particular dexterity and motor skills needed for magic would be a natural addition to this list; since these skills differ from those of other kinds of expert, the results would likely be of interest.

For example, Cavina-Pratesi et al. (2011) investigated pantomime movements of magicians. While normal people are generally quite poor at faking grasping, the fake movements of magicians were indistinguishable from real ones, suggesting that extensive practice results in different visuomotor—and possibly even visuospatial—mechanisms. Another useful skill is control of hand-eye co-ordination. In everyday life we tend to look at whatever we are manipulating (Hayhoe et al., 2003; Land, 2006). But because attentional misdirection often depends on the active manipulation of gaze (Kuhn and Land, 2006; Kuhn et al., 2009), magicians must learn to decouple eyes and actions. An interesting issue is the extent to which such decoupling can be achieved.

More generally, it would be interesting to explore the motor skills of magicians in the same way that skills are studied in other domains, such as sports (Land and McLeod, 2000) or music (Furneaux and Land, 1999). Since magicians learn their skills in a variety of ways (books, videos, personal training), there is also potential in examining how the style of learning affects skill development. To date, however, surprisingly few researchers have utilized this highly specialized and potentially valuable population.

#### Social aspects of expertise

In a related vein, it may also be worth using magicians to investigate the social aspects of the development of expertise. Most domains—such as sports or music—have formal educational resources in which expertise is developed. Magic is unusual in that there are few formal ways in which it can be learnt (i.e., few formal magic schools). Although the advent of social media has changed things to some extent, magicians are still generally reluctant to share their secrets with non-magicians, creating additional challenges. However, Rissanen et al. (2013) interviewed over a hundred professional magicians about the social network within which this expertise develops; results showed an interesting set of informal, yet intricate master–student relationships. Thus, the study of expertise in magic could provide a useful way to explore how specialized and secretive knowledge is shared^1^.

#### Magic and therapy

In recent years there has been considerable interest in using magic techniques as therapeutic tools (see Harte and Spencer, 2014). For example, most traditional therapies for children with hemiplegic cerebral palsy require repetitive and laborious actions, reducing compliance. But because children are keenly interested in learning magic tricks, therapeutic approaches involving the learning of sleight of hand result in significantly improved motor skills (Green et al., 2013). Magic has likewise been used as a therapeutic tool in pediatric counseling (Bowman, 1986), mental health (Lyons and Menolotto, 1990), psychotherapy (Moskowitz, 1973), and dentistry (Peretz and Gluck, 2005). A better scientific understanding of magic techniques might also help develop therapeutic tools in many other domains (e.g., social phobias, autism).

## NATURE OF MAGIC EFFECTS

Another set of approaches focuses not on the use of magic to study other phenomena, but on the nature of the magic effects themselves. Many effects can be produced only—or at least, far more effectively—via magic; as such, these could lead to issues of various kinds. Since these effects often push our perceptual and cognitive processes to their limits, the results could be highly illuminating.

### MAGICAL THINKING

An important part of magic is that its effects appear inexplicable; indeed, magic is sometimes defined as “creating illusions of the impossible” (Nelms, 1969/1996, p. 1). Such inexplicability could help us understand various aspects of cognition, such as the formation of belief systems. Whereas adults are generally skeptical, children tend to have a rich fantasy life with many magical elements—e.g., a belief in supernatural beings (Rosengren and Hickling, 1994). Such *magical thinking* is thought to play an important role in the development of cognition, in which “precausal” and magical explanations of the world are gradually replaced by causal ones (Piaget, 1927; Laurendeau and Pinard, 1962).

Although work on this issue has traditionally been based on the spontaneous explanation of everyday events, Subbotsky (2010) used a “magical box” that allowed the experimenter—unbeknownst to the observer—to switch objects (e.g., a stamp becoming a driver’s license). Most older children deny that magic can happen in the real world. However, when presented with the magical box they were just as likely to use magical as well as physical explanations (Subbotsky, 1997).

Most adults likewise deny the existence of real magic (Zusne and Jones, 1982). However, one study presented adults with a magical box into which the experimenter placed a plastic card; after casting a spell, the card was shown to have become badly scratched. Participants did not believe the scratches were caused by the spell. However, when asked to put their own hand in the device, most asked the experimenter not to cast the spell (Subbotsky, 2001). In another study, simple conjuring tricks portrayed as a demonstration of genuine psychic ability were found to enhance people’s beliefs in the paranormal (Mohr et al., 2014; see also Benassi et al., 1980). Such experiments are wonderful examples of how magic tricks can help study the formation of beliefs, and possibly superstitions. Indeed, such studies might even help distinguish between different kinds (or levels) of believability. For example, Lamont (2013) showed that people can believe in some apparently impossible things while not believing in others, or believe that an apparently impossible event actually occurred but not believe the explanation offered for it. It is also worth mentioning that some magicians consider a separation to exist between intellectual and emotional belief when seemingly impossible phenomena are encountered (e.g., Ortiz, 1994).

The results of such studies may have important clinical implications. For example, correlations appear to exist between magical thinking and obsessive-compulsive behavior (Bolton et al., 2002; Evans et al., 2002). And schizophrenic patients similarly appear to engage in a greater amount of magical thinking (Tissot and Burnand, 1980).

### THE EXPERIENCE OF WONDER

A central part of magic is the experience of wonder stemming from perceiving an event that is apparently impossible. Such phenomena can lead to humor, amazement, and surprise; they can even generate a sense of the laws of physics or logic being defied. Experiential states of this kind are difficult or even impossible to create in any other way.

It may be worth emphasizing that a magical experience does not occur simply from everyday reality being distorted. In a film, for example, a superhero can appear to fly across the sky. But when watching the film an explanation is readily available: special effects. Thus, although such effects are interesting, they are not inexplicable^2^. Indeed, if the spectator has an explanation for a trick—regardless of whether this explanation is true or not—the sense of wonder diminishes to some extent. Seeing a good magic trick creates a dilemma, a conflict between what the spectator thinks of as possible and the event that has been experienced. The more convinced the spectator is that the event cannot happen, the more powerful the effect, and the stronger the sense of wonder. Even if the observer does not believe in magic, there is still a split second in which reality is suspended, and wonder exists.

Experiential states such as wonder likely relate to our ability to distinguish between the possible and the impossible; this in turn may relate to how we learn to understand reality. Parris et al. (2009) had participants watch magic tricks while their brain activities were measured using fMRI. The areas activated were similar to those activated when experiencing impossible events such as violations in causality. Given that the failure to recognize the impossible is a likely foundation of psychotic disorders such as schizophrenia, such results might also lead to insights into the neurobiology of psychotic experiences.

Another potentially important contribution involves individual differences. Although magic is a universal art form, responses to it vary considerably. Some find it thrilling and exciting; others, irritating or even terrifying. Some are highly susceptible to magic; others, highly resistant. Individual differences exist in magical thinking (Subbotsky, 2004; Subbotsky and Quinteros, 2002), and it would be worth exploring whether similar differences exist in regards to other aspects of magic; they might reveal interesting personality traits, or cognitive or perceptual styles. For example, Kuhn et al. (2010) found that individuals with autism were more susceptible to the Vanishing Ball illusion, and had more problems in using gaze cues to allocate attention quickly enough to particular locations. Another interesting possibility is that—given the association of wonder with a child-like state of mind—a person’s childhood may affect the extent to which they experience wonder in a magic performance.

Finally, there is the possibility of better understanding wonder itself. Are different types of wonder created by different kinds of tricks? (e.g., viewing an apparent violation of object constancy vs. a mind-reading trick.) Is the sense of wonder created by an apparently inexplicable event comparable to that created by viewing a beautiful natural vista? All of these are interesting and important directions for future research.

## INVESTIGATION OF MAGIC TRICKS

Although the two main aspects of magic tricks—methods and effects—are individually useful for studying the human mind, additional insights can sometimes be obtained by considering them together—i.e., considering magic tricks as objects of scientific investigation in their own right. Any given trick involves various perceptual and cognitive mechanisms, in a context that includes factors such as the emotions of the spectator and the personality exhibited by the magician (see, e.g., Fitzkee, 1943/1988; Ortiz, 1994). Its study—usually in the form of a controlled experiment—therefore cuts across interesting issues in an interesting way. When controlled appropriately, such studies can rigorously establish that an effect exists (e.g., that forcing works under a given set of conditions) or that particular properties of the performance are relevant (and to what extent). With a bit of luck, these may even enable the underlying mechanisms to be mapped out.

### DECOMPOSITION

To explain a particular trick, magicians typically use informal principles of various kinds (e.g., Sharpe, 1988; Maskelyne and Devant, 1911/1992; Lamont and Wiseman, 1999). But more rigorous forms of investigation are also possible. Since a given magic trick has only one effect and one method, it is possible to focus on their interaction with some hope that relatively few mechanisms are involved. In addition, it is often possible to focus on just one *component* of a trick, and to simplify it so as to reduce the number of factors involved.

Decomposing a phenomenon of interest into simpler parts is an important part of scientific investigation. To see how this might proceed for a magic trick, consider what will be called here “The Materializing Card,*”* a variation of a commonly used trick based on forcing (Erdnase, 1902/1995). Here, the spectator is shown a deck of cards riﬄed quickly in front of them; they are asked to name a card as these cards flip by, after which the magician produces this card from a pocket, amazing the spectator (and the rest of the audience). This trick can therefore be decomposed into a sequence of components—seeing the card riﬄe and having a particular card come to mind, followed by seeing it in the magician’s pocket and experiencing a feeling of wonder. The first of these involves issues familiar to researchers in vision science (the actual seeing of the riﬄe), but also the forcing of a particular target card (caused by viewing the sequence). The second component involves seeing external reality align with the spectator’s choice—what might be called an *alignment effect*—followed by the sense of wonder evoked by that alignment. Each component might be considered as a minimal magic phenomenon. Indeed, such components might often be better candidates for investigation that complete tricks.

Decomposition simplifies analysis, and allows effort to be focused on those phenomena of greatest interest. But finding an appropriate decomposition is something of an art, requiring a “feel” for the matter at hand. The knowledge and experience of magicians would therefore be of great assistance here.

### ABSTRACTION

For a magician, an adequate description of a trick must contain enough detail about the method to enable its effect to be reproduced. Ideally, such a *concrete* description would also be enough to distinguish it from others, and give some idea about the particular circumstances—including theatrical setup—under which it is most effective. However, controlled investigation requires a version of the trick less concerned with the circumstances of a particular performance, and more with the general factors that influence the observer’s perceptual and cognitive mechanisms. For such an *abstract* trick (or component), the effect must be complex enough to still be interesting, while simple enough to allow behavior to be mapped out and explanations tested in a rigorous way. Interestingly, studies by magicians into principles of magic also involve considerable abstraction (e.g., de Ascanio, 1964; Sharpe, 1988); this would be another natural point of connection between scientists and magicians.

To see how abstraction might proceed, consider the forcing component of the Materializing Card. When a magician does this, various factors are at play, including the particular cards used, the story told, and the physical characteristics of the magician’s hands. But by focusing, say, only on the duration the cards are shown and their visibility, other details can be discarded, or at least made irrelevant. The result is a simpler, more abstract method (or *procedure*) involving just a few *key basefactors* that can be controlled in a straightforward way (Olson et al., 2013).

Ideally, the description of a procedure would include not only the key factors, but also a specification of how their values influence the strength of the effect. Mapping out such a specification would of course take work, but could be done in principle. For example, each of the 52 playing cards commonly used in magic tricks has been carefully measured in terms of properties such as visibility, memorability, and likeability (Olson et al., 2012). Subsequent studies on forcing, say, could determine whether or not these properties capture all the relevant attributes of a card, and how the value of each property (e.g., the level of visibility of the target card) affects the degree of forcing found.

Careful—and often quantitative—descriptive techniques were essential to the development of a scientific approach to areas such as chemistry (Dear, 2006, chap. 3). Similar considerations may apply here. For instance, the careful measurement of perceptual and cognitive characteristics of cards resulted not only in groupings that were known to magicians, but also in some that were not (Olson et al., 2012). Careful measurement based on abstract tricks has likewise revealed previously unknown factors influencing susceptibility to the Vanishing Ball illusion (Triplett, 1900; Kuhn and Land, 2006), and the inability to perceive rotary motion in the paddle move (Hergovich et al., 2011).

Finally, it may be worth pointing out that the abstract nature of a procedure provides an important *middle way* to connect the study of magic with its practice. The particular details of a performance are not critical for scientific purposes: what is important are the key factors manipulated, not the particular ways they are controlled. A practitioner’s technique can therefore inform scientific study while remaining secret, just as knowledge about an industrial process can be published in a useful abstract form (a patent, say) while hiding the proprietary details about its operation.

### EXPLANATION

As in the case of other phenomena involving perception or cognition, the explanation of a magic trick can be sought at three distinct levels of analysis: (a) the psychological mechanisms involved, (b) the neural implementation of these, and (c) the functional considerations (or computational theory) as to why these mechanisms are as they are. Only when explanation is achieved at all three levels can such a phenomenon be considered completely understood (Marr, 1982; Dennett, 1994; Glennerster, 2002).

#### Psychological mechanisms

A natural place to begin the explanation of a trick (or component) is with the *psychological mechanisms* involved—i.e., the functional mechanisms (perceptual and cognitive) that give rise to the observed behavior and subjective experience. There is no need here to specify how these mechanisms are grounded in the human nervous system, although neural plausibility is always welcome.

Because of its involvement with known psychological mechanisms, this level of analysis can sometimes enable new perspectives on old issues. For example, connections have been drawn between attentional misdirection and inattentional blindness (e.g., Kuhn and Tatler, 2005; Kuhn and Findlay, 2010), and between misdirection and change blindness (e.g., Rensink, 2000); indeed, strong links seem to exist between misdirection and attention research generally (Memmert, 2010; Memmert and Furley, 2010; Moran and Brady, 2010; Kuhn and Tatler, 2011). Such links have been used to support the three-network model of attention (Demacheva et al., 2012). They have even led to new perspectives—e.g., the proposal of two different types of inattentional blindness (Most, 2010). Interestingly, such developments have only become possible in the context of recent theories of visual perception, which emphasize the attentional factors involved in conscious visual experience (see, e.g., Rensink, 2010, 2015).

#### Neural mechanisms

In addition to psychological mechanisms, explanation can also appeal to the neural systems involved (see also Macknik and Martinez-Condé, 2009). This involves a *reduction* to elements of an entirely different kind—an explanation not in terms of the information-processing strategies of particular mechanisms, but in terms of the hardware used. Such reduction is rarely a single-step endeavor. An important step—and worthwhile goal in its own right—is *redescription:* establishing a non-causal link between a given trick and a set of neural mechanisms (i.e., neural correlates). For example, Parris et al. (2009) investigated the neural basis of seeing violations of causality in a magic effect. Here, circuits in the left dorso-lateral prefrontal and left anterior cingulate cortices were strongly activated, consistent with previous findings that these structures are recruited in situations involving cognitive conflict. A new discovery was that the activations associated with the violations were located in the left hemisphere, pointing to that hemisphere’s role in perceiving complex actions and events.

Although such results are important, it should be noted that the finding of neural mechanisms is only part of a much larger enterprise. It has been argued that “the perception of magic tricks will be best understood from a neurobiological perspective” (Macknik and Martinez-Condé, 2009, p. 241). In this view, a trick must be explained primarily in terms of neural mechanisms: psychological considerations have lower status^3^. But problems can arise if the search for neural mechanisms is considered the *primary* goal of scientific activity. As has been learned by other sciences concerned with human experience, a direct “jump” from consciously experienced effect to neural mechanism not only ignores important aspects of the processes involved, but also stands in danger of going astray, in that no checks are available from other levels of explanation.

#### Functional/computational considerations

Explanation in terms of mechanisms—both psychological and neural—can help us understand a given magic trick. But such understanding may still be incomplete. For instance, why do we even have a sense of wonder in the first place? Which circumstances invoke it? What kinds of violations give rise to what kinds of wonder? What—if anything—does this experience enable us to do?

Such issues are the concern of a *functional* (or computational) level of analysis, which focuses not only on describing the function carried out, but also on justifying *why* it has the form it has. In the case of wonder, for example, this experience may motivate the observer to think more about events that cannot be accounted for by the existing set of beliefs. An important observation in this regard is that spectators generally wish to see a trick repeated—not just to experience the effect again (which could be done via a different method), but to see how it was created in the first place. This points to the sense of wonder being connected to a strong need to understand what is going on. If so, the interesting possibility arises that the sense of wonder so essential to magic may also have been essential to the development of science.

In summary, then, explanation of magic tricks at all three levels of analysis could lead to interesting new insights into the nature of the human mind. Such analysis may not always be possible. But given the power of this approach even when it is only partially applicable (Dennett, 1994; Glennerster, 2002), it would appear worthwhile to at least attempt it in this domain.

## ORGANIZATION OF KNOWLEDGE

In addition to studying individual tricks and components, important insights might also be found by studying the *relationships* between them—e.g., natural groupings of tricks, or the set of methods that can create an effect. The study of such relationships is currently the least-developed way of using magic to study the human mind. However, if it can be sufficiently developed, it may become an important area of study that could connect in a productive way with other areas of research.

### INVENTORY

When organizing knowledge, a foundational issue is that of *description*. Although often linked to explanation (if only to clarify what is involved), description can proceed independently of this. Indeed, in sciences such as biology, structures are often described to a considerable extent without any real commitment to underlying causes (Mayr, 1982).

In many areas of study, description takes the form of an *inventory—*a complete listing of the entities under consideration (e.g., the set of known animals, or known songs). In the case of magic, such entities are clearly individual tricks, either concrete or abstract. Books that teach magic (e.g., Hay, 1947/1975; Nelms, 1969/1996) generally contain partial inventories, describing various tricks of interest. Early attempts toward a comprehensive set include that of Triplett (1900), who compiled a listing of many of the better-known tricks; these were described from the point of the performer and were loosely grouped, e.g., tricks involving optical illusions, or tricks involving unusual abilities. Later attempts include the work of Wright (1924), the collections of Fitzkee (1943/1988, 1945/1987), and Ortiz (1994), as well as the tricks in the taxonomies of Sharpe (1985, 1988) and Lamont and Wiseman (1999).

**FIGURE 2:**
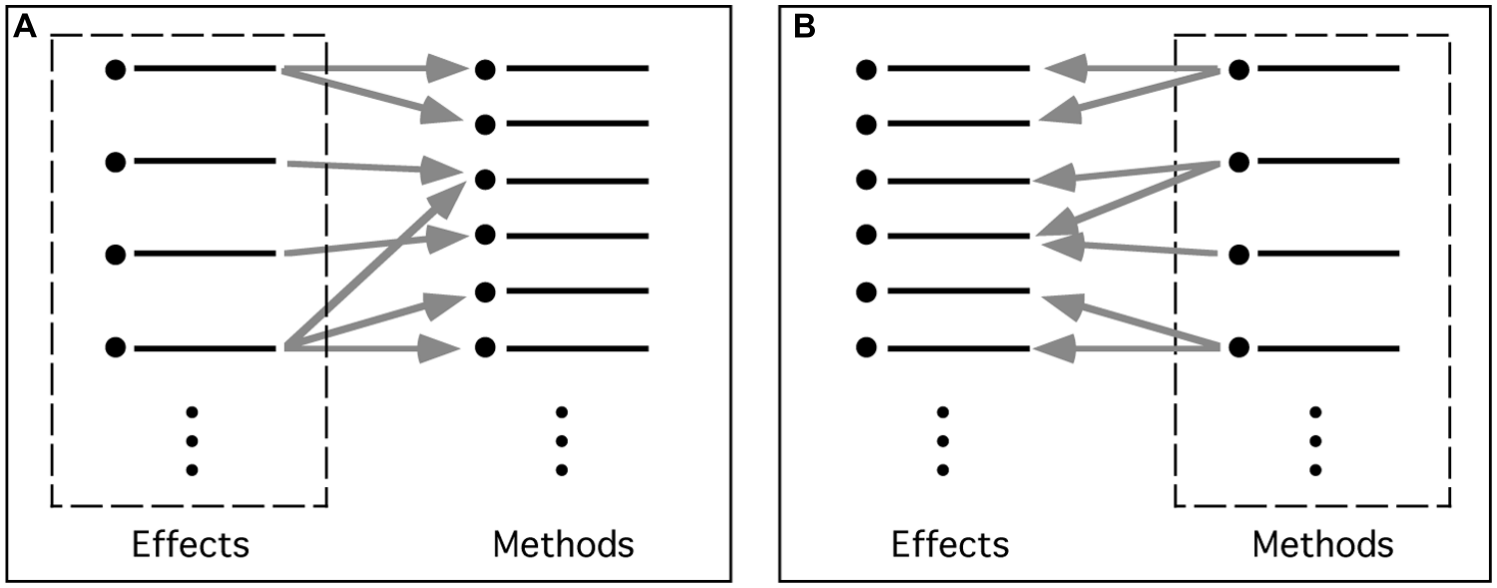
**Inventories centered on different aspects of magic tricks. (A)** Effect-centered inventory. Here, effects are primary, with each effect—or component—linked to the various methods able to create it. **(B)** Method-centered inventory. Here, each method—or component—is primary, and is linked to the set of effects that it helps create.

Strictly speaking, no particular organizational scheme (taxonomy) is required for an inventory. But what *is* required is that the many-to-many relationships between effects and methods should be maintained. One way of doing so is to have separate (although related) inventories centered on each aspect: one for the methods associated with each effect, and the other for the effects associated with each method (**Figure 2**). In the interests of simplicity, discussion here will focus on effect-centered inventories.

Such inventories could be of two kinds. A *concrete inventory* describes concrete tricks; it is essentially a record of magic practice, ensuring that all known effects and methods are accessible to the community of practitioners. An example of this is the website “Ask Alexander,”^4^ an on-line library containing descriptions of literally millions of concrete magic tricks. An *abstract inventory* could likewise describe all known abstract tricks (or components)—i.e., abstract effects along with the procedures for producing them (see Abstraction). Such an inventory could form much of the basis for scientific work.

### TAXONOMY

Although usually considered part of an inventory, a distinct level of description can be separated out: that of *taxonomy*. Its main goal is to organize tricks via particular kinds of relationships, including a set of “pattern elements” that could generate any patterns found among these relationships. Taxonomic developments have been critical to the scientific development of several fields—e.g., biology, chemistry, and mineralogy (see, e.g., Dear, 2006). They would likely play a similar role here. As in the case of inventories, taxonomies could be concrete or abstract, and effect-centered or method-centered. Since each has different perspectives, all these kinds would likely be useful in supporting scientific study.

Various taxonomies have been proposed over the years (e.g., Triplett, 1900; Wright, 1924; Bruno, 1978; Lamont and Wiseman, 1999). None, however, has received overwhelming acceptance (Lamont et al., 2010). These schemes are largely folk taxonomies, similar to the groupings used by naturalists in early classifications of animals, or the tables of chemical affinities used prior to modern chemistry^5^. Interestingly, doubt also existed long ago as to whether natural—or even adequate—taxonomies could exist in those domains (e.g., Dear, 2006, chap. 2). But just as folk taxonomies in those areas eventually gave way to natural taxonomies, a similar development might be possible here. Note that although more knowledge always helps, a complete understanding of magic is not necessary for this—for example, new taxonomies continue to appear in various subdomains of vision science (Changizi, 2009; Gregory, 2009) even though our scientific understanding of visual perception remains incomplete.

One way of developing better-founded taxonomies might be to start with the magician’s language and conceptual schemes, and make these clearer and more rigorous (Kuhn et al., 2008; Kuhn, 2010). This would likely involve various subtle distinctions. Consider, for instance, the term “misdirection.” This is a broad concept, referring to any manipulation that directs the spectator away from the cause of an effect (see Cognition). In the case of visual attention, it might be defined as a “deflection of attention for the purpose of disguise” (Sharpe, 1988, p. 47), which would seem sufficient for most purposes. But various issues still remain. For example, it has been suggested (Lamont et al., 2010) that distraction of the type typically used in scientific experiments has little to do with the misdirection used in magic. But while misdirection is indeed more than distraction, it nevertheless is still related—for example, the use of gaze by magicians to direct attention away from a method is similar to the use of gaze to control attention in scientific studies (e.g., Friesen and Kingstone, 1998; Kuhn and Kingstone, 2009). A final resolution of this issue will probably be difficult, but the outcome may well improve our understanding of the issues involved. This will likely be the case for other terms as well.

A somewhat related approach would be to reconsider the features used as the basis of classification: a wider range of features might be used, say, or more quantitative measures. The *principles* of organization might also be made more quantitative and methodical—e.g., assigning different weights to different properties. (For an interesting account of this approach in biology, see Yoon, 2009).

Another way of developing more natural taxonomies might be to base them on established psychological mechanisms and principles. For example, a taxonomy of misdirection (and thus, much of magic) can be created via two objective taxonomic principles: (i) base it as much as possible on known psychological mechanisms, and (ii) have the highest levels be based on the mechanisms affected, followed by the mechanisms that control them (Kuhn et al., 2014). Such a taxonomy relies on the nature of these mechanisms—and their relationships to each other—to lessen the subjective element in its organization. A possible complication could arise if a particular trick affects more than one mechanism. But this could be handled by making the component—rather than the complete trick—the basic element of the taxonomy^6^. Indeed, this approach would have the added benefit that the variations of a trick would not need to be considered as separate entities in the taxonomy, but as related combinations of similar components (cf. molecules vs. atoms in chemistry^7^).

### A SCIENCE OF MAGIC?

Given that different kinds of knowledge about magic can help investigate the human mind, questions arise about the extent to which this could be done. Could the study of magic be carried out in a coherent way that encompasses most magic tricks? Could it eventually become an area of research akin to, say, vision science, resulting in a better understanding of known effects, and perhaps even the prediction of new ones?

In what follows, we present a few—admittedly incomplete—suggestions about how this issue might be approached. These proposals are necessarily tentative. But our intent here is to show that there does exist some possibility of organizing a study of magic as a scientific discipline, one that could enable a better understanding of magic tricks, and ultimately, a better understanding of human perception and cognition.

#### Scope

Sciences of many kinds exist. Some, such as physics, have considerable theoretical structure; others, such as meteorology, far less. Some, such as biochemistry, have a strong experimental component; others, such as geology, rely on natural observation. But all involve a *process* of inquiry, a particular way of thinking about issues. In particular, all sciences have a clearly defined set of entities in the world considered relevant, and a set of issues concerning these entities. The set of entities selected—the *scope*—is critical for the success of this enterprise: if too broad, the discipline will lose coherence—e.g., the original science of vision in Hellenistic times, which included mathematical geometry, physical optics, and physiological considerations. If too narrow, the result will be a set of unnatural divisions or an insufficient “critical mass” of basic concepts. Given these considerations, what might be the proper scope for a possible science of magic?

One choice might simply be the set of effects and methods currently used by magicians. But the particular tricks in current use is only a partial set of those possible; their selection is largely due to arbitrary factors such as prevailing fashion. Consequently, systematic connections may not always exist between them. Moreover, this set is time-bound: it is not the same as what was used in the past, nor will it likely be the same as what will be used in the future (Lamont, 2013).

Another choice might be the ways that humans can be deceived. This avoids a direct dependence on the tricks in current use while still capturing much of what happens in a magic performance; indeed, magic is sometimes characterized this way (e.g., Hyman, 1989; Triplett, 1900). But deception can take a very wide variety of forms, ranging from fiction to advertising to counterfeiting to psychological warfare to simple everyday lying. As such, it risks incoherence. Even more importantly, it misses the main point of magic: people do not attend magic shows simply to be deceived.

What to do? We propose that a more natural focus is *the experience of wonder generated by perceiving an apparently impossible phenomenon* (cf. see The Experience of Wonder). This experience appears to be common to all effects considered “magical,” no matter what they involve, or when or where they occur. Moreover, this characterization is a positive one, with magic defined not in terms of the failure of a mechanism (as occurs in deception), but in terms of a positive experience. In this view, the scope of scientific investigation into magic would be any aspect of any phenomenon associated with this experience. This focus is not limited to the set of magic tricks in current use; instead it concerns the resulting experiential state and any possible technique that could produce it, both of which are timeless^8^. It also emphasizes the experience of wonder—an experience that has not received much serious investigation to date—and makes it the central concern, which then lends coherence to the entire enterprise.

#### Framework

A clearly defined scope is necessary for any area of science. But it is also helpful to have a research framework—a coherent set of characterizations, issues, and practices to help guide research and assess how a given work contributes to it (cf. Lakatos, 1978). What might this look like for the case of magic?

**FIGURE 3:**
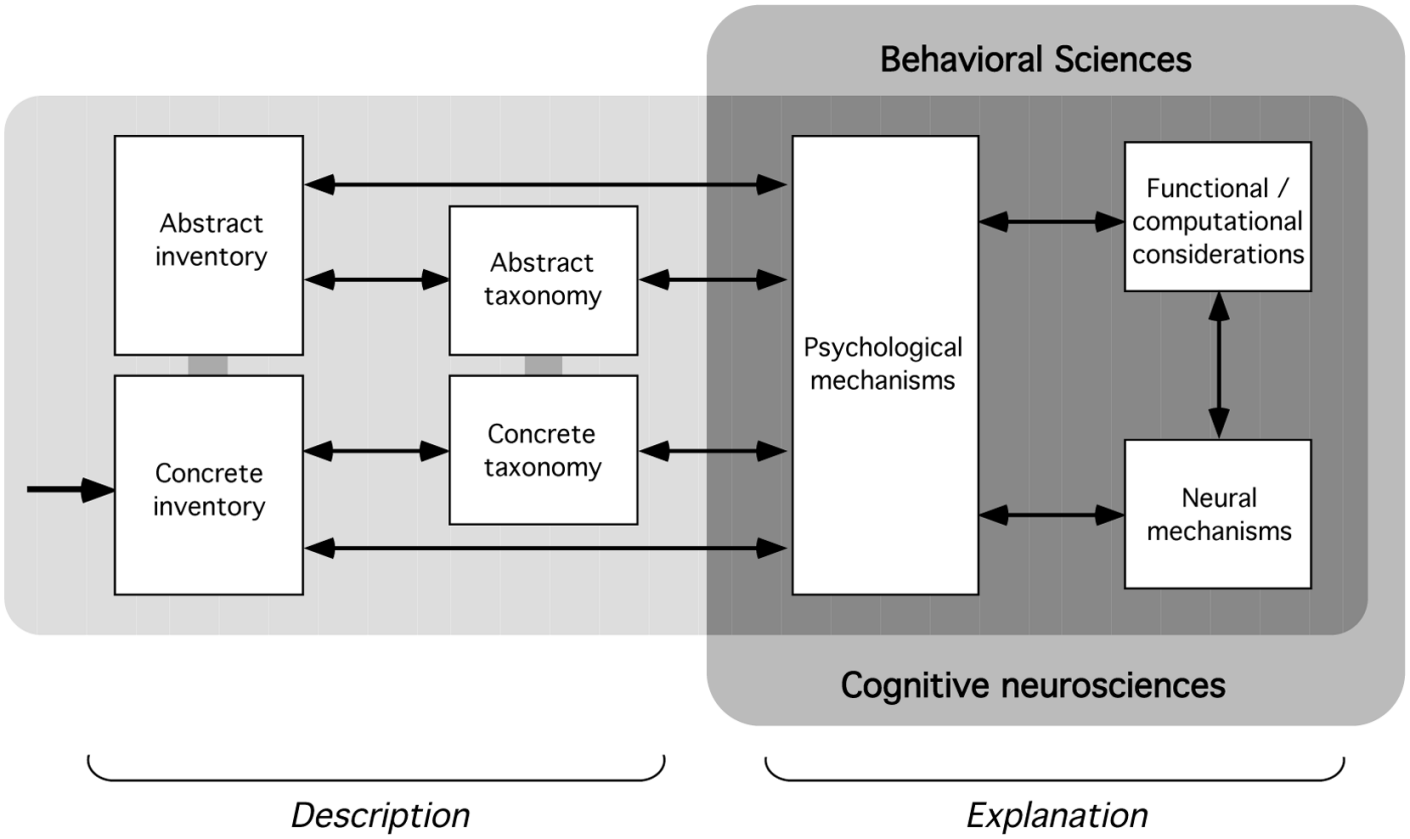
**Framework for a possible science of magic.** Each part concerns a particular set of related issues. The arrow indicates that investigation would begin with the collection of individual tricks the concrete inventory; these could be abstracted and collected into an abstract inventory (see Inventory), and eventually organized into a concrete or abstract taxonomy (see Taxonomy). Analysis of the individual tricks (or components) in the inventories and the patterns of relationships in the taxonomies would proceed in terms of their psychological, neural, and computational mechanisms (see Explanation).

One possibility is shown in **Figure 3**, which largely incorporates suggestions made earlier in this paper. It can be divided into two groups of issues: those concerning *description* (issues of inventory and taxonomy), and those concerning *explanation* (analysis in terms of psychological mechanisms, neural mechanisms, and computational theory). The descriptive parts would supply material for explanation; these could be developed as sketched in sections “Inventory” and “Taxonomy.” Explanation of these would proceed along the lines sketched in section “Explanation,” with analysis carried out at three different levels (psychological, neural, and functional).

An important application of this would be to find deep patterns or principles underlying the explanation of many magic tricks (or components). Such commonalities could point to mechanisms that are not apparent when investigating individual tricks or the relatively superficial patterns in the taxonomy. A search for general principles common to individual mechanical devices appears to have helped create the science of mechanics (Berryman, 2009), which was then able to connect to other sciences; if mechanical techniques and effects have parallels to magical ones, some possibility exists of a similar development here.

The ultimate argument for or against a possible science of magic, however, will be the extent to which it can uncover new knowledge and produce interesting new effects. We do not claim that this enterprise will necessarily succeed; there may well be obstacles of which we are currently unaware. But at the moment nothing appears to stand in its way. And nothing ventured, nothing gained.

## APPLICATIONS TO MAGIC PRACTICE

As many magicians have pointed out over the years (e.g., Houdin, 1868/2006; Wonder and Minch, 1996), a better understanding of the perceptual and cognitive mechanisms underlying various aspects of magic could well inform the design of better magic tricks, and perhaps even presentation techniques. The relation between applied and basic science is a reciprocal one: just as the insights obtained in an applied area can provide subject matter for the corresponding basic science, so can the lessons learned in an abstract science be applied to concrete concerns (Stokes, 1997). Such transfer has long been the case in various domains (e.g., using knowledge of biochemistry to help design more effective medications). There appear to be no *a priori* reasons why such transfer could not also occur here.

This need not be limited to human performance. Interaction with computers can be an important part of various magic tricks (Marshall et al., 2010). And given the complexities involved in human-computer interaction, knowledge of particular effects or methods could inform the design of more effective computer interfaces, creating a more compelling “user illusion” (Tognazzini, 1993). Such knowledge might even suggest ways to enable the computer itself to control a user’s expectations or attention, leading to the development of “magical displays” that could capture some aspects of the performance of a human magician (Rensink, 2002). There may also be interesting connections with special effects. For example, the creation of pixie dust that is perceived as “magical” is extremely difficult to achieve using computer graphics; it seems to rely in part on the dust appearing natural, but still not ordinary (Gilland, 2009). Knowledge about what makes something appear magical (and why) would be most helpful in creating effects of this kind.

## CONCLUSION

We have proposed here a framework describing various ways in which knowledge of magic can help contribute to the understanding of the human mind. These are grouped into four distinct levels: (i) using known methods as the basis of new methodologies, (ii) using known effects to explore new aspects of the mind, (iii) investigating how particular tricks (suitably abstracted) relate to psychological and neural mechanisms, and (iv) studying the patterns of relationships between individual tricks (and perhaps their components). Among other things, this framework suggests the possibility of an organized body of study—perhaps even a science—centered around the sense of wonder that is experienced when encountering an apparently impossible event.

The prospects for this enterprise appear to be good. Magicians can manipulate our perception and cognition in powerful and consistent ways, and have noticed enough structure and systematicity to propose various categorizations. Our role as scientists is to ask the right questions and use the right methods to investigate this further, and make this area as rigorous and systematic as possible. Similar attempts are underway for other performing arts: work has started on a psychology of music with comparable goals (see, e.g., Levitin, 2007), and similar efforts are also being attempted for film (e.g., Shimamura, 2013; Smith, 2014). It will be interesting to see the extent to which the developments in these domains converge with those for magic.

In this context, it should be mentioned that many aspects of magic not discussed here are also worthy of scientific investigation—e.g., the character of the magician (Fitzkee, 1943/1988; Ortiz, 1994), the use of ritual (Sorensen, 2006), or the use of conjuring principles by psychic mediums (Marks, 2000). These issues are clearly beyond the scope of what is proposed here. Our goal in this paper is a more modest one: simply to determine the viability of a “core” area of study, including some of the steps needed to carry it out in practice. The success of this will ultimately depend on the willingness of researchers from a wide range of disciplines to link some of their own investigations to this endeavor.

The eventual identity of this area of inquiry is difficult to ascertain. It might become a loose network of related results in various fields. It might become part of an existing science—e.g., an area of “magic perception” in vision science similar to, say, scene perception, or it might become part of the psychology of emotion. If valued for its insights into connections that cut across various issues, it might develop a more autonomous identity—e.g., a “psychology of wonder” or “psychology of magic” similar in status to say, social psychology, with connections to the study of perception and cognition, but keeping its own traditions and set of core research issues. Only time will tell. But, however, events unfold, it appears that the study of magic has sufficient focus and coherence to prevent it from dissolving into a set of disconnected studies in disconnected fields.

Magic is an ancient art form centered around wonder and surprise. As such, its practice relies on a level of secrecy that needs to be respected. In recent years, the possibility of a science of magic has received public as well as scientific attention. Part of the reason for this is that magic offers an engaging and entertaining way to illustrate and discuss complex psychological theories, thereby providing a valuable educational tool. Although public interest is valuable for science, there is also danger of revealing sensitive details, and thus damaging this wonderful art. As we have argued above, there exists a “middle way” that keeps secret the details of concrete implementations but still allows public and scientific discussion of general principles. We strongly encourage researchers in this field to use such an approach, and so maximize the likelihood that people will continue to experience all the wonder and amazement that magic offers.

## Conflict of Interest Statement

The authors declare that the research was conducted in the absence of any commercial or financial relationships that could be construed as a potential conflict of interest.

## Footnotes

1 There is an interesting parallel with the scientific community, which is uses a similar mentoring system to train new practitioners, and which—at least in its phases prior to publication—often maintains a degree of secrecy. Both communities also honor members who make significant contributions (often after years of work), and look down upon members who steal ideas.

2 The earliest audiences of the cinema often reported feelings of astonishment and wonder, similar to those experienced in magic, likely because no explanation was readily available for the amazing transformations they saw (Gunning, 1989). As audiences began to understand the mechanisms involved, such reports vanished.

3 To be sure, Macknik et al. (2008) do mention behavior on occasion. But consider: in the main body of Macknik et al. (2008), the word “neural” is used 30 times, whereas the word “behavioral” is used only twice—once at the beginning of the article and once at the end, both uses being simple glosses.

4 http://askalexander.org/

5 The term “folk” does not imply that a taxonomy is inaccurate—for example, the traditional folk taxonomy for animals (as developed in the West, anyway) is often not far from the scientifically based Linnaean one. Rather, it simply describes *how* the taxonomy was created: via utilitarian principles generated on the basis of social knowledge, vs. the more context-free approach typical of science. Although valuable, traditional magic taxonomies don’t have a scientific basis—it is difficult, for example, to imagine a discovery that could make such a taxonomy change its structure.

6 If misdirection is viewed as the component of a trick that hides the method generating the “main” effect experienced, this is exactly what is done in the taxonomy of Kuhn et al. (2014). If this applies more generally, magic tricks might best be described via a set of such taxonomies, each pertaining to a particular component.

7 Interestingly, the magician Fitzkee (1944/1989) proposed that all effects could be constructed out of 19 “basic effects,” and proposed a formula by which new effects could be generated. It might be worth revisiting this, given the better understanding of perceptual and cognitive mechanisms than was available in 1944.

8 In classical antiquity, several forms of entertainment were reported as creating a sense of wonder and delight, via mechanical devices that appeared to start (and continue) on their own. Central to this was ensuring that the causes of the movements could not be readily determined, and that the effects violated the expectations of the spectator (Berryman, 2009, pp. 140–142, 175–176). Although these might not be considered as forms of magic according to traditional views, they would be so here.
